# Editorial: Emerging advances in exploiting pulmonary administration for treatment of thoracic diseases

**DOI:** 10.3389/fbioe.2025.1612172

**Published:** 2025-04-25

**Authors:** Lina Wu, Gregory M. Lanza, Jin Xie, Dipanjan Pan

**Affiliations:** ^1^ Molecular Imaging Research Center, the Fourth affiliated hospital of Harbin Medical University, Harbin, China; ^2^ School of Medicine, Washington University in St. Louis, St. Louis, MO, United States; ^3^ Department of Chemistry, University of Georgia, Athens, GA, United States; ^4^ Department of Materials Science and Engineering, The Pennsylvania State University, University Park, PA, United States

**Keywords:** pulmonary administration, thoracic diseases, lung cancer, IPF, artificial intelligence

## Introduction

Thoracic diseases represent some of the major healthcare challenges worldwide. This field has witnessed remarkable advancements in recent years, with demonstrated efficacy in managing respiratory diseases such as asthma, chronic obstructive pulmonary disease (COPD), and cystic fibrosis (Bhatt et al., 2023; Woodward and Fromen, 2024). Encouragingly, emerging applications extend to respiratory infections, pulmonary oncology, and even systemic disorders including diabetes, revealing broader therapeutic potential (Li et al., 2022). Despite these advances, still some challenges remain to attract wide attention from academic and industrial circles. This Research Topic, titled “Emerging Advances in Exploiting Pulmonary Administration for Treatment of Thoracic Diseases,” aimed to address the latest research in this field, including clinical trials, formulations, biosafety, Artificial Intelligence (AI) applications et al., through pulmonary administration by compiling cutting-edge research.

**SCHEME 1:**
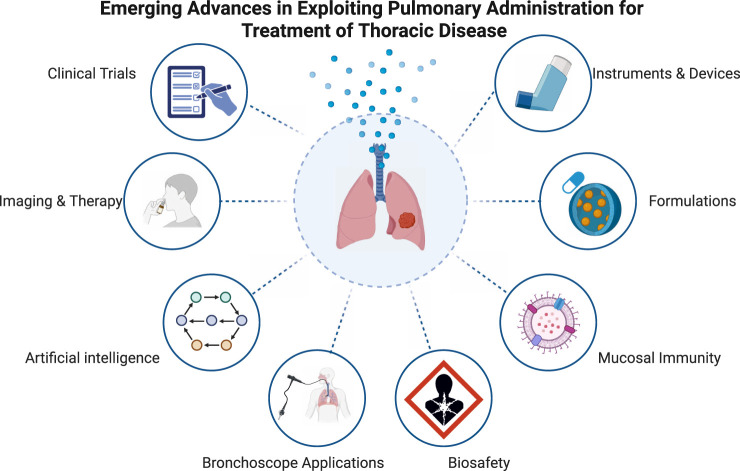
The solicited submissions scope of this Research Topic (This image is generated by Biorender).

## Scope and contributions

In this Research Topic, we solicit submissions reporting recent progress on pulmonary administration. Related topics include but were not limited to the following: Clinical trials on new drugs delivered through the pulmonary route. Investigating nanoparticle or genetic formulations given through pulmonary administration. Advanced aerosol, nebulization, instillation, or inhalation therapies. Safety and pharmacokinetics-related studies. Mechanisms of imaging and therapy. Intratracheal or pulmonary delivery instrument, device, or medical facilities. Delivery-associated complications. Bronchoscope applications in the context of thoracic disease treatment. Artificial Intelligence applications in precision detection of thoracic diseases and challenges in the pulmonary delivery of therapeutics, Scheme 1.

Focusing on these subjects, this Research Topic received 15 submissions, of which 4 were accepted (27% acceptance rate). Key contributions include: A kind of dual-modality MRI/fluorescence ultrasmall iron oxide nanoprobe was successfully synthesized that can effectively assess and dynamically monitor atherosclerotic plaques, showing potential for clinical translation (Zhang et al.). A retrospective study on the characteristics of respiratory muscle function and influencing factors in patients with dyspnea and normal or mildly abnormal lung function (Yang et al.). A novel method for precise implantation of a tracheal Y-shaped stent (Ding et al.) and a review report on the development of clinical trials for non-small cell lung cancer drugs in China from 2005 to 2023 (Jia et al.).

## Future perspectives

The field of pulmonary administration is evolving rapidly, particularly in the treatment of localized lung diseases, driven by the innovations in nanotechnology, advanced aerosol systems, and gene therapy (Wang et al., 2023). Nevertheless, critical challenges still need to be addressed to translate promising preclinical innovations into successful clinical application. Key priorities include improving alveolar targeting precision to minimize proximal airway drug loss and overcoming bioavailability limitations linked to pulmonary deposition variability and metabolic clearance (Wang et al., 2024; Yang et al., 2022). Ensuring nanocarrier safety and developing scalable production methods for complex systems (e.g., lipid nanoparticles, exosomes) remain vital for clinical adoption. Interdisciplinary approaches—such as AI-optimized carrier design, patient-tailored therapies based on anatomical and biomarker data, and strategies to bypass pulmonary barriers (e.g., mucus clearance, epithelial junctions) (Li et al., 2025; Liu et al., 2024; Ren et al., 2025). It can be anticipated that targeted pulmonary therapies will continue to serve as pivotal contributors to global respiratory disease management, driven by rapid interdisciplinary advancements in precision medicine.

## References

[B1] Bhatt S. P. Vogelmeier C. F. Cole J. Bafadhel M. Rabe K. F. Hanania N. A. (2023). Dupilumab for COPD with Dupilumab for COPD with Type 2 Inflammation Indicated by Eosinophil Countsype 2 inflammation indicated by eosinophil counts. N. Engl. J. Med. 389 (3), 205–214. 10.1056/NEJMoa2303951 37272521

[B2] Li Y. Yang J. Gu G. Guo X. He C. Sun J. (2022). Pulmonary delivery of theranostic nanoclusters for lung cancer ferroptosis with enhanced chemodynamic/radiation synergistic therapy. Nano Lett. 22 (3), 963–972. 10.1021/acs.nanolett.1c03786 35073699

[B3] Li Z. Guo Z. Zhang F. Sun L. Luan H. Fang Z. (2025). Inhalable biohybrid microrobots: a non-invasive approach for lung treatment. Nat. Commun. 16 (1), 666. 10.1038/s41467-025-56032-4 39809831 PMC11733022

[B4] Liu S. Wen Y. Shan X. Ma X. Yang C. Cheng X. (2024). Charge-assisted stabilization of lipid nanoparticles enables inhaled mRNA delivery for mucosal vaccination. Nat. Commun. 15 (1), 9471. 10.1038/s41467-024-53914-x 39488531 PMC11531489

[B5] Ren F. Aliper A. Chen J. Zhao H. Rao S. Kuppe C. (2025). A small-molecule TNIK inhibitor targets fibrosis in preclinical and clinical models. Nat. Biotechnol. 43 (1), 63–75. 10.1038/s41587-024-02143-0 38459338 PMC11738990

[B6] Wang B. Xiang J. He B. Tan S. Zhou W. (2023). Enhancing bioavailability of natural extracts for nutritional applications through dry powder inhalers (DPI) spray drying: technological advancements and future directions. Front. Nutr. 10, 1190912. 10.3389/fnut.2023.1190912 37476406 PMC10354342

[B7] Wang W. Zhong Z. Huang Z. Hiew T. N. Huang Y. Wu C. (2024). Nanomedicines for targeted pulmonary delivery: receptor-mediated strategy and alternatives. Nanoscale 16 (6), 2820–2833. 10.1039/d3nr05487j 38289362

[B8] Woodward I. R. Fromen C. A. (2024). Recent developments in aerosol pulmonary drug delivery: new technologies, new cargos, and new targets. Annu. Rev. Biomed. Eng. 26 (1), 307–330. 10.1146/annurev-bioeng-110122-010848 38424089 PMC11222059

[B9] Yang J. Li Y. Sun J. Zou H. Sun Y. Luo J. (2022). An osimertinib-perfluorocarbon nanoemulsion with excellent targeted therapeutic efficacy in non-small cell lung cancer: achieving intratracheal and intravenous administration. ACS Nano 16 (8), 12590–12605. 10.1021/acsnano.2c04159 35863049

